# Systematic Review and Meta-Analysis of Human Skin Diseases Due to Particulate Matter

**DOI:** 10.3390/ijerph14121458

**Published:** 2017-11-25

**Authors:** Le Thi Nhu Ngoc, Duckshin Park, Yongil Lee, Young-Chul Lee

**Affiliations:** 1 Department of BioNano Technology, Gachon University, 1342 Seongnam, Korea; nhungocle92@gmail.com; 2 Korea Railroad Research Institute (KRRI), 176 Cheoldobakmulkwan-ro, Uiwang-si 16105, Korea; dspark@krri.re.kr (D.P.); freego83@krri.re.kr (Y.L.)

**Keywords:** particulate matter (PM), PM_10_, PM_2.5_, meta-analysis, human skin diseases

## Abstract

This study investigated the effects of particulate matter (PM) on human skin diseases by conducting a systematic review of existing literature and performing a meta-analysis. It considered articles reporting an original effect of PM on human skin. From among 918 articles identified, 13 articles were included for further consideration after manual screening of the articles resulted in the exclusion of articles that did not contain data, review articles, editorials, and also articles in languages other than English. Random-effects models and forest plots were used to estimate the effect of PM on the skin by Meta-Disc analysis. According to people’s reports of exposure and negative skin effects (atopic dermatitis (AD), eczema, and skin aging, etc.) due to air pollution, the summary relative risk (odds ratio) of PM_10_ was determined to be 0.99 (95% confidence interval (CI) 0.89–1.11) whereas PM_2.5_ was determined to be 1.04 (95% CI 0.96–1.12). Simultaneously, there was a different extent of impact between PM_10_ and PM_2.5_ on atopic dermatitis (AD) for those of young age: the odds ratio of PM_10_ and PM_2.5_ were 0.96 (95% CI 0.83–1.11; I^2^ = 62.7%) and 1.05 (95% CI 0.95–1.16; I^2^ = 46%), respectively. Furthermore, the results suggest an estimated increase of disease incidence per 10 μg/m^3^ PM of 1.01% (0.08–2.05) due to PM_10_ and 1.60% (0.45–2.82) due to PM_2.5_. Following the results, PM_10_ and PM_2.5_ are associated with increased risks of human skin diseases, especially AD, whose risk is higher in infants and school children. With its smaller size and a high concentration of metals, PM_2.5_ is more closely related to AD in younger people, compared to PM_10_.

## 1. Introduction

Air pollution in both outdoor and indoor environments is a longstanding worldwide issue. Among air pollutants, the most commonly monitored are particulate matter (PM), nitrogen dioxide (NO_2_), sulfur dioxide (SO_2_), and ozone (O_3_). According to the annual report of the World Health Organization (WHO) titled State of Global Air 2017, over 90% of the world’s population live in areas with unhealthy air, which is a leading risk factor for diseases and death [1]. The WHO has established that premature death by air pollution occurs as the direct results of cardiovascular diseases, respiratory diseases, and lung cancer at rates of 80%, 14%, and 6%, respectively [2].

One of the most common components of air pollution is PM, which is classified as PM_10_, fine PM, and ultrafine particles according to the particles’ aerodynamic diameter [3]. PM_10_ (particles of less than 10 μm diameter) is composed of particles from dust, industrial emissions, and traffic emissions; such inhalation of PM_10_ is directly related to various respiratory diseases [4,5,6]. A smaller PM diameter with less than 2.5 μm is defined as fine PM (PM_2.5_); PM_2.5_ is primarily comprised of organic carbon compounds, nitrates, and sulfates [4]. Recently, ambient PM_2.5_ has become increasingly present in the surrounding air and significantly involved in human health, particularly in regard to respiratory tract diseases, as it can reach the bronchial tubes and deep regions of the lung [7]. Reference cohort studies in which participants are monitored for decades have discovered that smaller particles such as PM_2.5_ have more adverse effects on human health than larger particles [4,8,9,10]. Additionally, epidemiological investigations into contamination, especially ambient air pollution, indicated that the PM is not only correlative with the exacerbation of cardiovascular diseases and respiratory systemic inflammation impacts but also the progression of inflammatory skin diseases [11] such as atopic dermatitis (AD) [12,13,14], acne, psoriasis, and allergic reactions [9,15,16,17].

Nowadays, more evidence is available on the effects of PM of various sizes (PM_10_ and PM_2.5_) on skin diseases (e.g., AD and eczema in children, cellulitis and skin aging in adult) [10,16,17,18]. The present study entailed a systematic review and meta-analysis by summarizing the statistically significant effects of PM on human skin and its association with multiple skin diseases and their symptoms.

## 2. Materials and Methods

### 2.1. Literature Search and Data Extraction

The literature search on the adverse skin effects of PM_10_ and PM_2.5_ air pollution on the adverse skin effects of PM_10_ and PM_2.5_ air pollution was performed in the English-language databases PubMed (National Library of Medicine, Bethesda, MD, USA), Elsevier (Information and Analytics, Amsterdam, The Netherlands), and Web of Science (Institute of Scientific Information and Clarivate Analytics, United States) and considered articles published between 1990 and 2017. Combinations of the following keywords were used: PM, PM_10_, PM_2.5_, human skin diseases, AD, skin aging, and eczema disease. Reference lists of identified papers were also searched.

Bibliographic reference lists were manually selected for meta-analysis based on identifying associations between PM_10_, PM_2.5_, and human skin diseases, articles that presented no data (e.g., review articles and editorials) as well as articles written in languages other than English were excluded. The inclusion criteria for quantitative meta-analysis were estimates of diseases reporting data that could be used to calculate an estimate of the effect.

From each of the selected studies, the title, author, location, publication year, study design, number of events, and specific risk estimates were extracted and entered into a Microsoft Excel database.

### 2.2. Meta-Analysis

The effect estimates from the selected studies were summarized using the inverse variance method, by which the overall effect estimate was the average of the individual study effect estimates that was weighted by the inverse of the study variance [19]. In our meta-analysis, first, each study’s heterogeneity was examined using the standard coefficient heterogeneity (I^2^) test. The existence of heterogeneity was considered at the 95% level of significance and I^2^; according to that result, either fixed-effects or random-effects models were used to assess the pooled estimates.

All analyses were performed using Meta-Disc software (version 1.4, Unit of Clinical Biostatistics, Marid, Spain). 

## 3. Results

**Figure 1:**
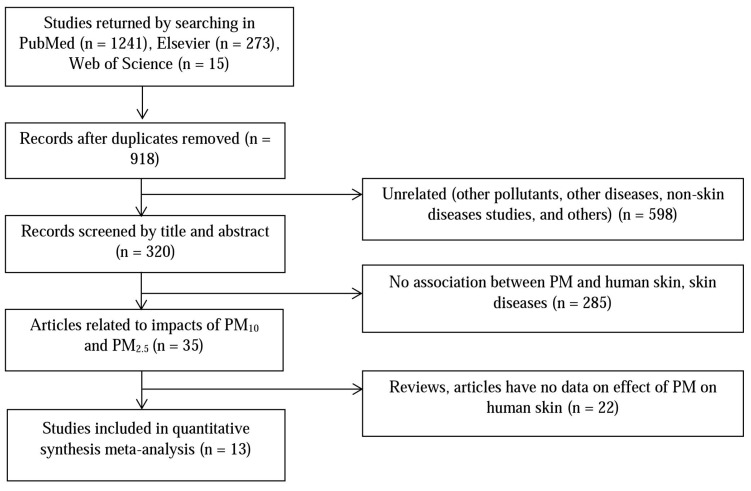
Systematic screening stage for literature review.

Figure 1 shows the study’s article selection process. The database searches yielded a total of 320 unique publications whose titles and abstracts were screened. After exclusion of articles not relevant to the human skin impacts of PM_10_ and PM_2.5_, or containing no pertinent data, 13 studies were included in the quantitative meta-analysis. Among them, there was a report of three skin diseases associated with PM [20]: pigment spots, wrinkles, and skin aging; one study described the effect of PM on two symptoms [16]: eczema and itchy rash; and the influences of both PM_10_ and PM_2.5_ on human skin were considered by three studies [21,22,23]. The 13 studies included 72,000 total participants, with school children and women representing almost all of the participants (see Table 1 for the study’s summary characteristic), and all of the studies provided raw data on the effect estimates.

**Figure 2:**
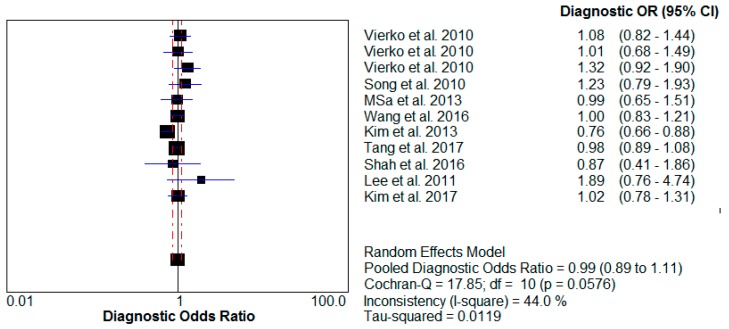
Relative risk of PM_10_ impact on human skin. OR: odds ratio.

**Figure 3:**
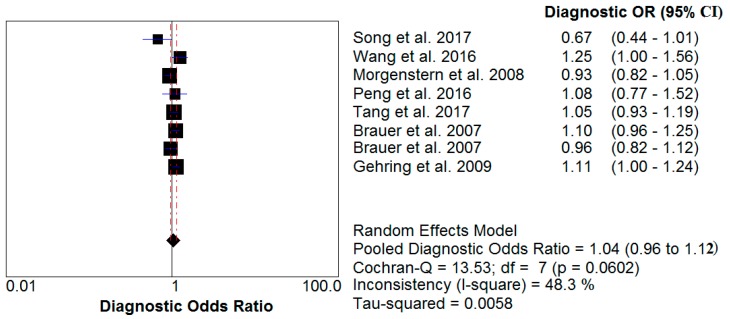
Relative risk of PM_2.5_ impact on human skin.

A meta-analysis of these studies yielded summary relative risks (odds ratio) of 0.99 (95% confidence interval (CI) 0.89–1.11) for PM_10_ impact and 1.04 (95% CI 0.96–1.12) for PM_2.5_ influence. The test for heterogeneity was not significant for either PM_10_ (I^2^ = 44% < 50%) or PM_2.5_ (I^2^ = 48.3% < 50%). The results of this analysis are provided (Table 2, Figure 2 and Figure 3).

Furthermore, to assess the impact of PM on the skin in those of a young age, particularly for AD disease, a sub-analysis of studies was performed that included the influence estimates for different sized PM. The results indicated that PM_2.5_ is directly related to AD in young people (Table 3), showing an odds ratio of 1.05 (95% CI 0.95–1.16) and coefficient heterogeneity (I^2^) of 46%; in contrast, the heterogeneity was significant for the PM_10_ effect, showing an odds ratio of 0.96 (95% CI 0.83–1.11) and I^2^ of 62.7% > 50%.

Estimates of the effects of short-term exposure to PM_10_ and PM_2.5_ were analyzed on the basis of increase incidence of skin diseases per 10 μg/m^3^ increase in PM_10_ and PM_2.5_. For each increase in PM_10_ and PM_2.5_ concentration, the risk of human skin diseases due to PM was determined to be 1.01% (0.08–2.05) and 1.60% (0.45–2.82), respectively. The results are presented in Table 4.

The relationship between concentrations of PM and human skin diseases over long-term exposure is presented in Table 5. The outcomes showed that when concentrations reach upwards of 47.09 μg/m^3^ for PM_10_ and 26.04 μg/m^3^ for PM_2.5_ human skin could be adversely affected.

## 4. Discussion

In this systematic review and meta-analysis of more than 46,100 cases of PM impact on human skin from 13 studies, this study confirmed that both PM_10_ and PM_2.5_ have a statistically significant impact on skin diseases. Moreover, referencing estimates of the WHO and studies related to the impacts of PM to health [32,33,34], PM not only causes usual skin diseases but may also lead to skin cancer (basal cell carcinoma and squamous cell carcinoma) [35] and other health issues (e.g., cardiovascular disease, respiratory system, and asthma [4,16]).

**Figure 4:**
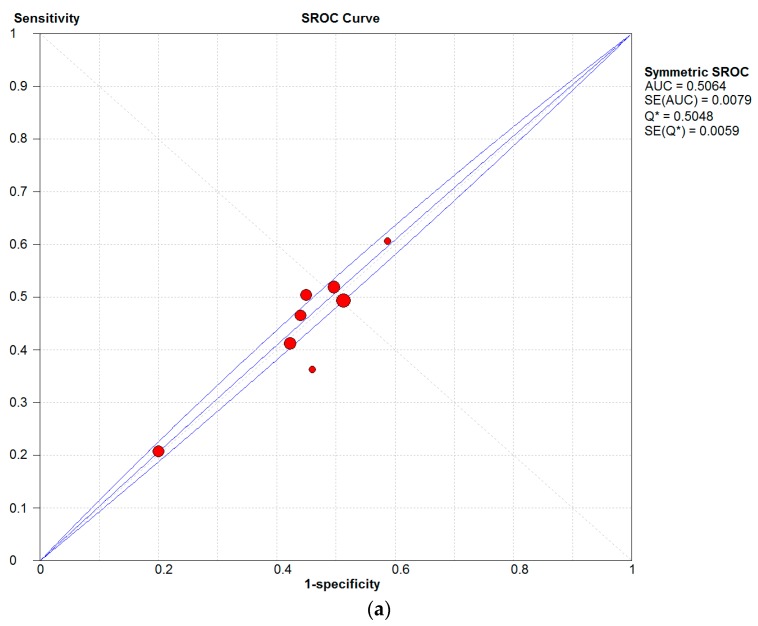

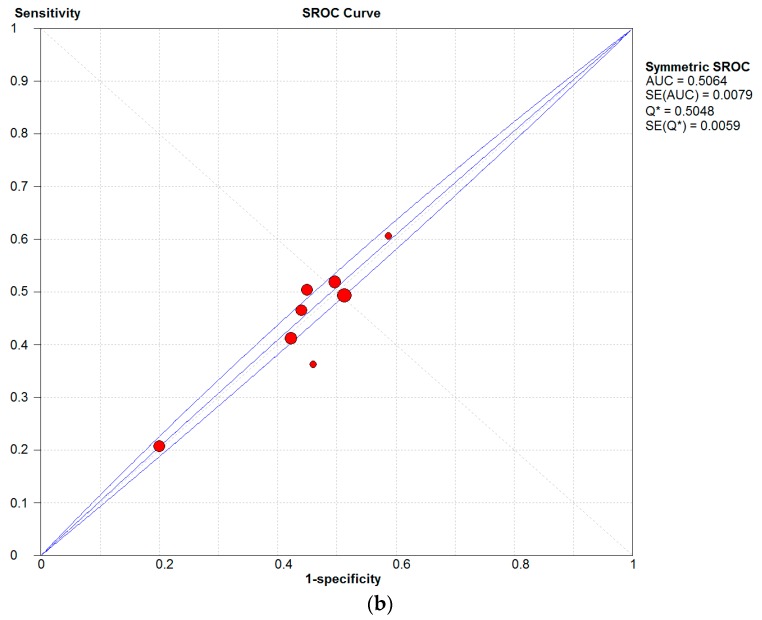
Summary receiver operating characteristic (SROC) curves of influences of (**a**) PM_2.5_, (**b**) PM_10_ on human skin.

In this study, it was found that PM is closely associated with AD, eczema, and skin allergies. In Figure 4, a high sensitivity can be observed for the influences of both PM types on human, which is compelling evidence of an association between air pollution and human skin diseases.

Furthermore, the results demonstrated that with each 10 μg/m^3^ increase, PM_10_ and PM_2.5_ increased disease incidences by 1.01% (0.08–2.05) and 1.60% (0.45–2.82), respectively (Table 4). The outcomes suggest that an increase in PM_2.5_ exposure concentration could slightly elevate the incidence of skin disease compared to PM_10_. PMs differ not only according to their varying physical and chemical characteristic but also their concentrations by location (e.g., components, sources, structure, surface, and diameter, etc.) [36,37]. In general, PM_10_ and PM_2.5_ include inhalable particles that are small enough to penetrate deep into skin and regions of the respiratory system, especially as a consequence of long-term exposure. Humans are at risk of a greater incidence of diseases due to lower PM_2.5_ concentrations (26.04 μg/m^3^) than PM_10_ concentrations (47.09 μg/m^3^); the results of this study indicated that PM_2.5_ was significantly more harmful to human health than PM_10_ [36].

Additionally, according to the results (Table 6), these estimates demonstrated a larger impact of PM to skin allergies, which demonstrated 54% sensitivity. Even though the estimates showed lower sensitivities for AD and eczema disease (26% and 47% sensitivity, respectively), there were more cases of these skin conditions than skin allergies, particularly among children and infants [16,29,31].

Most of the subjects in all of the studies were young (2–30 years old), including newborns, children, and adolescents. Indeed, the skin of individuals in these age groups is sensitive, resulting in a higher likelihood of effects due to exposure to air pollution. Long-term exposure to air-pollution sources (e.g., smoking, PM, NO_2_, SO_2_, etc.) in the home, outdoors, and at school contributes to many health problems such as wheezing and asthma as well as cardiovascular and skin diseases (e.g., cellulitis, skin itching, itchy aging, AD, etc.) [17,26,31,38].

The coefficient heterogeneity (I^2^ = 46%) from the results of the meta-analysis demonstrated the presence of high concentrations of PM_2.5_ in the air, which was one of the direct causes of AD in the younger age groups, particularly newborns and children. Furthermore, heterogeneity existed for the influence of PM_10_ (I^2^ = 62.7% > 50%), but it is not easy to include or exclude the causal effect of PM_10_ on AD diseases, as more research is needed in order to obtain better statistical evidence and an enhanced understanding of that possible association. In particular, higher contents of cadmium, copper, lead, nickel, vanadium, and zinc in PM_2.5_ were associated with increased eczema prevalence and AD [38], and the ratio of heavy metals in PM was more abundant in PM_2.5_ than in PM_10_ [39], thus contributing towards making PM_2.5_ potentially more harmful to humans than PM_10_, specifically via oxidative stress. Therefore, the next standard will have to focus on smaller particles that are more likely to be responsible for adverse health effects.

## 5. Conclusions

Observationally, PM is one of the most common components of air pollution. There is evidence that metals in PM cause DNA, skin-cell, and protein damage as well as apoptosis through the mitochondria-regulated death pathway [39]. PM_10_ and PM_2.5_ in high concentrations can promote the development and exacerbation of various skin diseases. Based on these meta-analysis results, it can be added that there are associations between PM_10_, PM_2.5_, and skin diseases, and furthermore, that there is an increased probability of PM-associated diseases at young ages.

The major differences between the two particulate fractions are in the number, concentration, and composition of the smallest particles [40]. PM_2.5_, with its smaller size and a larger number of component metals, can easily penetrate deep into skin cells, and, as such, can pose a higher risk of AD disease than PM_10_; PM_2.5_ thus has a major role in adverse impacts of air pollution on human health [41]. Therefore, PM_2.5_ might be more closely associated with PM-induced skin diseases.

Even though PM has general diameter and mass concentration standards associated with skin diseases in humans, PM has varying physical and chemical characteristics, hence monitoring of PM_10_ and PM_2.5_ needs to be improved in many countries to asses population exposure and to assist local authorities in establishing plans for improving air quality (limits for emissions from various sources, reducing energy consumption, and changing modes of transport, etc.) [36] so that we can control not only human skin diseases but also many serious diseases (e.g., lung cancer, cardiovascular diseases, and respiratory diseases, etc.) due to PM exposure.

## 6. Limitations of Study

A limitation of this study is the fact that it included only observational, cohort studies, and individual studies and only small quantities of those; therefore, we could not clearly delineate the relationships among PM, air pollution, and human skin diseases. In the future, if there are cohort studies and/or case-control studies about the impact of PM on skin human diseases, more significance and greater confidence can be placed on determining the degree of impact from different relevant variables.

## Figures and Tables

**Table 1:** Summary characteristic of studies. AD: atopic dermatitis; PM: particulate matter.

Reference	Year of Study	Location	Pollutant	Diagnosis	Total No.	Age
Vierko et al., 2010 [20]	2008–2009	Europe	PM_10_	Pigment spots, wrinkles, skin aging	400	>18
Song et al., 2011 [23]	2009	South Korea	PM_10_, PM_2.5_	Skin itching	670	8–12
Peng et al., 2016 [24]	2015	China	PM_2.5_	Skin itching	611	10–15
MSa et al., 2013 [25]	2011	France	PM_10_	Current eczema	518	10–12
Wang et al., 2015 [21]	2010	China	PM_10_	AD	5925	8–15
Morgenstern et al., 2008 [26]	2005	Germany	PM_2.5_	Eczema	10,750	4–6
Kim et al., 2013 [27]	2009–2010	South Korea	PM_10_	AD	1880	8–12
Tang et al., 2017 [22]	2011	Taiwan	PM_10_, PM_2.5_	AD	5115	20–30
Shah et al., 2016 [28]	2015	United States	PM_10_	Eczema	128	28–30
Brauer et al., 2007 [16]	2004–2006	Germany	PM_2.5_	Eczema, itchy rash	6982	3–6
Lee et al., 2011 [29]	2010	South Korea	PM_10_	AD	51	2–3
Gehring et al., 2009 [30]	1996–2000	Netherlands	PM_2.5_	Allergen	3863	8
Kim et al., 2017 [31]	2013–2014	South Korea	PM_10_	AD	35,158	4–8

**Table 2:** Sizes of studies in primary meta-analysis. CI: confidence interval.

Reference	Odds Ratio (95% (CI))		Diagnosis
	PM_10_	PM_2.5_	
Vierko et al., 2010 [20]	1.08 (0.82–1.44)		Pigment spots
	1.01 (0.68–1.49)		Wrinkles
	1.32 (0.93–1.90)		Skin aging
Song et al., 2011 [23]	1.23 (0.79–1.93)	0.67 (0.44–1.01)	Skin itching
Peng et al., 2016 [24]		1.08 (0.77–1.52)	Skin itching
MSa et al., 2016 [25]	0.99 (0.65–1.51)		Current eczema
Wang et al., 2015 [21]	1.00 (0.83–1.21)	1.25 (1.00–1.56)	AD
Morgenstern et al., 2008 [26]		0.93 (0.82–1.05)	Eczema
Kim et al., 2013 [27]	0.76 (0.66–0.88)		AD
Tang et al., 2017 [22]	0.98 (0.89–1.08)	1.05 (0.93–1.19)	AD
Shah et al., 2016 [28]	0.87 (0.41–1.86)		Eczema
Brauer et al., 2007 [16]		1.10 (0.96–1.25)	Eczema
		0.96 (0.82–1.12)	Itchy rash
Lee et al., 2011 [29]	1.89 (0.76–4.74)		AD
Gehring et al., 2009 [30]		1.11 (1.00–1.24)	Allergen
Kim et al., 2017 [31]	1.02 (0.78–1.31)		AD

**Table 3:** Impact of PM on AD * for those young age.

Reference	Odds Ratio (95% CI)	
	PM_10_	PM_2.5_
Song et al., 2011 [23]	1.23 (0.79–1.93)	0.67 (0.44–1.01)
Wang et al., 2015 [21]	1.00 (0.83–1.21)	1.25 (1.00–1.56)
Peng et al., 2016 [24]		1.08 (0.77–1.52)
Kim et al., 2013 [27]	0.76 (0.66–0.88)	
Tang et al., 2017 [22]	0.98 (0.89–1.08)	1.05 (0.93–1.19)
Brauer et al., 2007 [16]		0.96 (0.82–1.12)
Lee et al., 2017 [29]	1.89 (0.76–4.74)	
Kim et al., 2017 [31]	1.02 (0.78–1.31)	
Gehring et al., 2009 [30]		1.11 (1.00–1.24)
Summary relative risk (95% CI)	0.96 (0.83–1.11)	1.05 (0.95–1.16)
The coefficient heterogeneity I^2^ (%)	62.7	46.0

* AD included AD, skin itching, general allergens, and itchy rash.

**Table 4:** ER (%) of skin diseases due to short-term exposure to PM.

Reference	Location	Diagnosis	ER (%) Skin Disease (95% CI)	
			PM_10_	PM_2.5_
Kim et al., 2013 [27]	South Korea	AD	0.44 (0.12–0.77)	0.67 (0.03–1.38)
Wang et al., 2015 [21]	China	AD		1.54 (1.03–2.32)
Kim et al., 2017 [31]	South Korea	AD	3.20 (1.50–4.90)	
Morgenstern et al., 2008 [26]	Europe	Eczema		1.00 (0.97–1.04)
Song et al., 2011 [23]	South Korea	Skin itching	1.03 (0.02–2.23)	3.10 (0.20–6.10)
Kim et al., 2017 [31]	South Korea	AD	0.36 (0.05–0.71)	
Msa et al., 2013 [25]	France	Eczema	1.02 (0.84–1.24)	
Seo et al., 2015 [9]	South Korea	AD	0.57 (0.23-0.98)	
Gehring et al., 2010 [30]	Netherlands	Allergen		1.68 (0.41–2.07)
Ahn Kangmo, 2015 [12]	South Korea	AD	0.44 (0.16–0.74)	
Combined estimate			1.01 (0.08–2.05)	1.60 (0.45–2.82)

ER (%): Excess risk: percent increase skin disease (95% CI) per 10μg/m^3^ increase in PM_10_ and PM_2.5_.

**Table 5:** Relationship between PM concentration and human skin diseases over long-term exposure.

Reference	Location	Diagnosis	Pollutant	
			PM_10_ (μg/m^3^)	PM_2.5_ (μg/m^3^)
Vierkotter et al., 2010 [20]	Germany	Skin aging		6.50
Gehring et al., 2010 [30]	Netherlands	Allergen		25.20
Kim et al., 2013 [27]	South Korea	AD	50.50	25.60
Peng et al., 2016 [24]	China	Skin itching		35.20
Wang et al., 2015 [21]	China	AD	48.32	29.07
Shah et al., 2016 [28]	United States	Eczema	56.26	
Kim et al., 2017 [31]	South Korea	AD	45.20	
Brauer et al., 2007 [16]	Netherlands	Eczema		25.20
Morgenstern et al., 2008 [26]	Europe	Eczema		15.13
Song et al., 2011 [23]	South Korea	Skin itching	44.89	22.38
Tang et al., 2016 [22]	Taiwan	AD	56.30	33.60
Msa et al., 2013 [25]	France	Eczema	31.00	
Seo et al., 2015 [9]	South Korea	AD	46.80	
Szyszkowicz et al., 2016 [17]	New York	Cellulitis		27.00
	Hamilton			33.50
	Halton			34.20
Combined estimate			47.09 (42.01–52.17)	26.04 (20.66–31.42)

**Table 6:** Summary sensitivity and specificity of AD, eczema disease, and skin allergies due to PM.

Category	No. of Studies	Summary Sensitivity, % (95% CI)	Summary Specificity, % (95% CI)	Q * (%)
AD	7	26.4 (25.5–27.4)	46.5 (46.1–46.9)	50.25
Eczema	4	47.2 (45.2–49.3)	50.5 (49.8–51.2)	50.09
Skin allergen	6	54.3 (51.3–57.3)	49.5 (47.7–51.3)	49.76

Q* corresponds to the point on the SROC curve where sensitivity and specificity are equal.
